# Intestinal thromboangiitis obliterans: a case report

**DOI:** 10.1186/s13256-021-02719-3

**Published:** 2021-04-23

**Authors:** Swastik Sourav Mishra, Tushar Subhadarshan Mishra, Suvradeep Mitra, Pankaj Kumar

**Affiliations:** 1 Department of Surgery, All India Institute of Medical Sciences, Bhubaneswar, Odisha India; 2 Department of Pathology, All India Institute of Medical Sciences, Bhubaneswar, Odisha India; 3 AIIMS Bhubaneswar, Room No. 403, Academic building, AIIMS Road, Patrapada, Bhubaneswar, Sijua 751019 India

**Keywords:** Acute mesenteric ischemia, Buerger’s disease, Smoking, Thromboangiitis obliterans, Case report

## Abstract

**Background:**

Thromboangiitis obliterans or Buerger’s disease is a form of peripheral vascular disease in young male smokers. The involvement of the intestine occurs in only about 2% of the cases, when they may present as acute abdomen due to mesenteric ischemia. The uncommonness of the condition makes it a less suspected differential diagnosis, leading to a delay in appropriate management, thereby increasing chances of morbidity or mortality. Cessation of smoking is known to stall the disease progression including visceral involvement, but may not always be the case as happened in the case being presented.

**Case presentation:**

Our Indian Hindu male patient, a known smoker, presented with diffuse abdominal pain along with bouts of vomiting and loose motions. He had a prior history of amputation of the right foot, 4 years before. At presentation he had abdominal distension with diffuse tenderness and guarding. An omental band attached to the tip of the appendix was discovered at the initial exploration along with dilated proximal bowel loops, for which a release of the omental band along with appendectomy was done. He developed an enterocutaneous fistula on the 6th postoperative day for which he had to be reexplored, and multiple jejunal perforations were found. Segmental jejunal resection and a Roux-en-Y gastrojejunostomy with distal ileostomy were done along with a feeding jejunostomy. The patient however again had feculent discharge from the wound for which a third exploration was done. The gastrojejunostomy and feeding jejunostomy sites were leaky, both of which were repaired primarily. The patient developed septicemia which progressed to refractory septic shock, and he ultimately succumbed to his illness on the 23rd postoperative day of the index surgery.

**Conclusion:**

Acute abdomen in a young man who is a chronic smoker and having an antecedent history of amputation of some part of an extremity for a nontraumatic cause should raise the suspicion of Buerger’s disease of the intestine. Although it is a progressive disease and the situation has already progressed by the time intestinal symptoms manifest, early detection may give some scope of salvage and decrease the morbidity and mortality.

## Background

Thromboangiitis obliterans (TAO) or Buerger’s disease by convention is considered a disease of the limbs, usually the lower, unless otherwise specified. Intestinal involvement is rare, seen only in 2% cases, when they may present with features of varying degrees of mesenteric ischemia, sometimes amounting to acute abdomen. A prior history of smoking and amputation in a case with clinical features of mesenteric ischemia should raise suspicion of Buerger’s disease of the intestine. However, the diagnosis may be missed unless there is a high index of clinical suspicion and due emphasis is given to this typical antecedent history, which is imperative for timely management. Impaired circulation, secondary to chronic narrowing of the mesenteric vasculature, manifests with postprandial abdominal pain and weight loss. On the other hand, acute onset of ischemia manifests as severe abdominal pain, with vomiting and blood-mixed stool, eventually progressing to features of peritonitis [1]. Early exploration is indispensable not only for resection of any necrotic bowel segment but also to rule out any focus of thrombus or emboli. CT angiography (CTA) is a useful initial investigation to demonstrate the narrowing of the vessels and ischemic changes in the gastrointestinal (GI) tract. However, histopathological characterization of the vessels in the resected bowel is diagnostic. The awareness of the histopathologists and the exclusion of atherosclerosis and other vasculitic disorders are essential for diagnosing this rare entity. This case is being presented to highlight how the failure to pick up the clinical clues early can prove costly leading to a delay in diagnosis and frittering away a possible chance of salvaging the patient.

## Case presentation

A 45-year-old non-hypertensive and non-diabetic Indian Hindu male presented to the casualty with pain in the abdomen for 10 days, along with vomiting and loose stools for 5 days. The pain was constant, diffuse, and severe, with an increase in its intensity for the last 3 days, and was associated with a non-bilious type of vomiting and loose stools. Besides, there was associated history of blood in stool for the previous 3 days. The patient had a history of smoking with the amputation of his right mid-foot 4 years prior to this admission, detailed records of which were not available. The patient claimed to have stopped smoking since the amputation. There was no history of alcohol consumption or any comorbidity. There was no family history of any similar illness.

On examination, the patient was afebrile, normotensive, conscious, alert, and cooperative. The pulse rate was 110 per minute. The abdomen was slightly distended with the presence of diffuse tenderness and guarding. The liver dullness was not obliterated, and the bowel sounds were sluggish. On the digital rectal examination, the rectum contained blood-stained fecal matter. Appendicular perforation peritonitis and an ischemic bowel pathology were kept as differentials (the possibility of Buerger’s disease of intestine was never suspected). The ultrasonography of the abdomen showed dilated small bowel loops. His complete blood count and liver and renal function tests were within normal limits. Serum antinuclear antibody (ANA) and anti-neutrophil cytoplasmic antibody (ANCA) tests were negative.

**Fig. 1:**
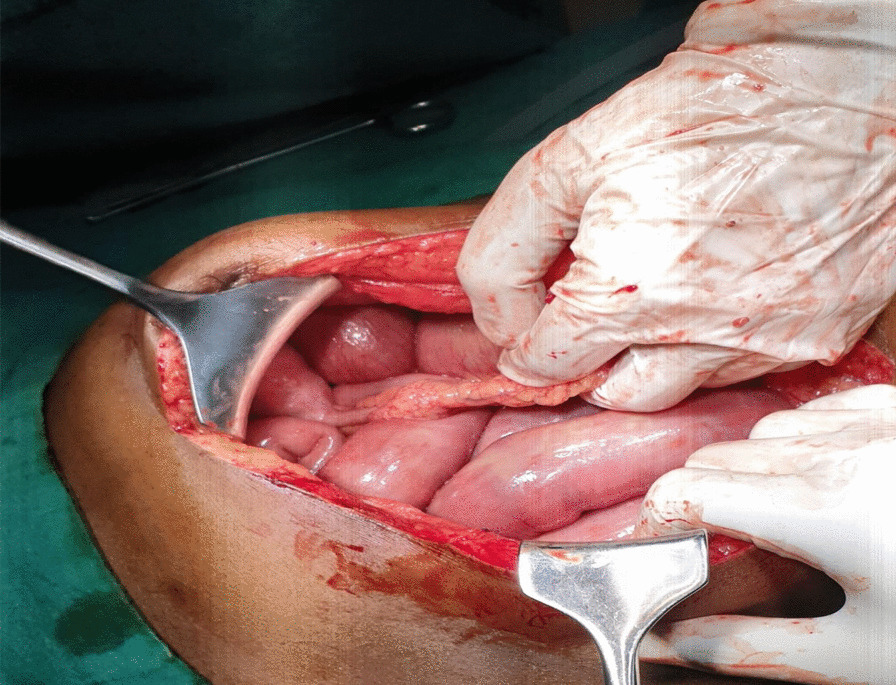
First exploration: band adherent to the appendix

**Fig. 2:**
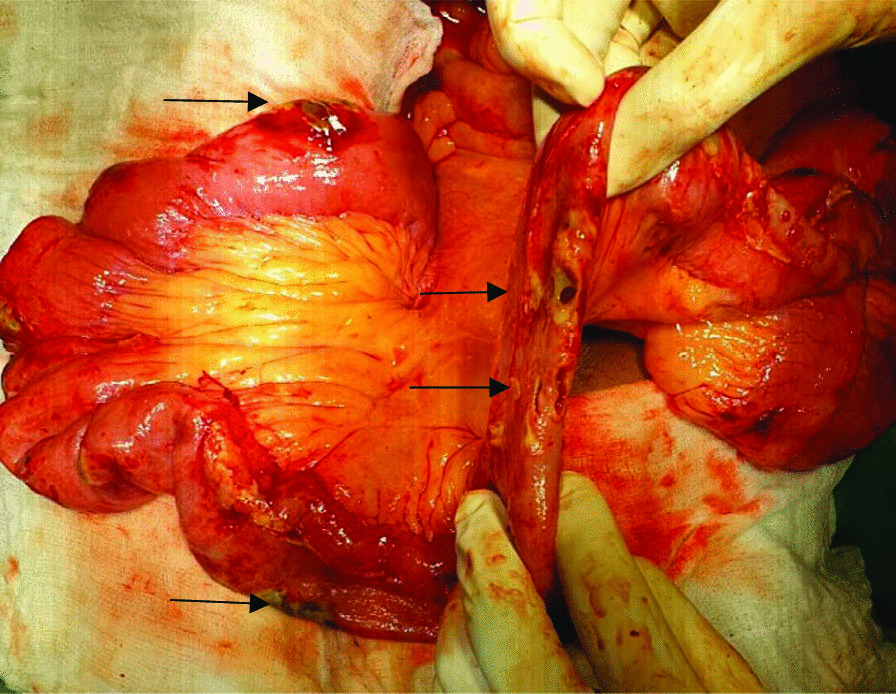
Black arrow showing multiple areas of perforation in the second exploration

**Fig. 3:**
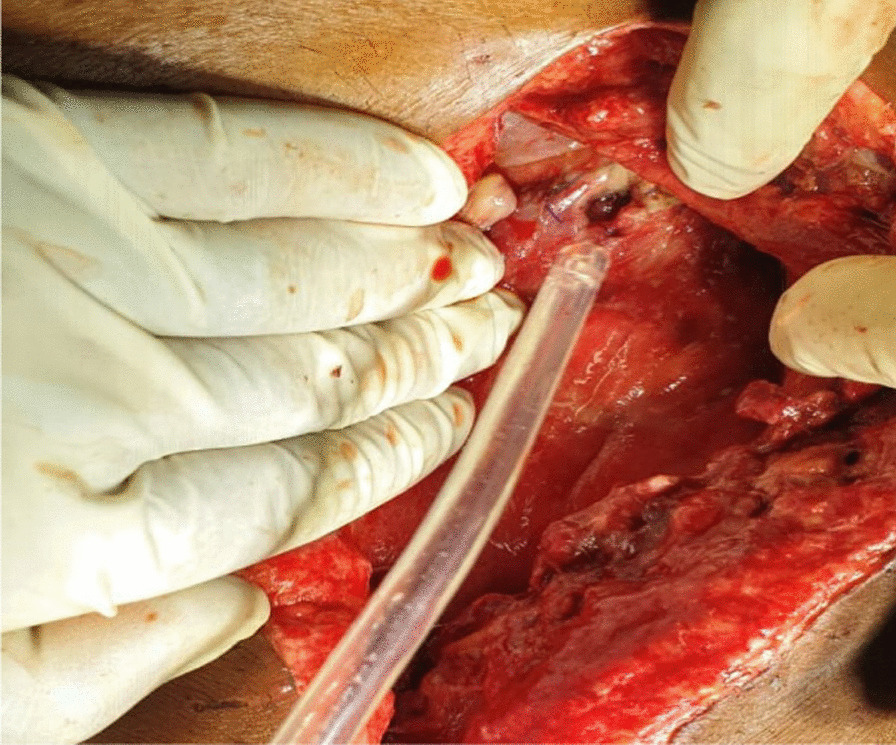
Leak from the gastrojejunostomy site in the third exploration

On exploratory laparotomy, an omental band was found adherent to the tip of the appendix (Fig 1). This appeared to be causing an intestinal obstruction as the proximal bowel loops were dilated. Hence, an appendicectomy along with the release of the appendico-omental band was done. The patient clinically improved initially for a few days postoperatively, when oral liquids were started. However, symptoms of vomiting and blood-mixed stool recurred, and the abdomen was tender. He started discharging pus from the midline abdominal wound from the 2nd postoperative day, for which the wound was laid open. On the 6th postoperative day, there was feculent discharge from the wound for which he was re-explored, which revealed multiple patchy areas of gangrene in the jejunum with perforations starting 5 cm distal to the duodenojejunal (DJ) flexure up to 60 cm distally. There were multiple jejunal perforations in this involved segment of the jejunum. In addition, there were two small perforations adjacent to each other approximately 10 cm proximal to the ileocecal junction (Fig. 2). Two feet of the affected jejunum was resected, and a Roux-en-Y gastrojejunostomy and distal loop ileostomy were performed along with a feeding jejunostomy. The patient again developed feculent discharge from the wound site on postoperative day 3 of the second surgery, for which another laparotomy was undertaken. The feeding jejunostomy and the gastrojejunostomy site were found to be leaking (Fig. 3). A primary repair of the feeding jejunostomy and a falciparum ligament patch repair of the gastrojejunostomy leak sites were performed. However, the patient developed septicemia with persistently elevated serum lactate levels, which progressed to refractory septic shock, and he ultimately succumbed to the illness on day 23 of the first surgery.

The appendicectomy and jejunal resection specimens from the first two laparotomies were sent to for histopathology examination. The jejunal segment measured 70 cm in length. It contained multiple patchy gangrenous areas along with perforation approximately at distances of 3 cm, 5 cm, 15 cm, 37 cm, 42 cm, 50 cm, and 66 cm from the proximal resection end. The corresponding mucosal surfaces showed transverse ulcers with bile staining and multiple transverse perforations at places. The largest ulcer measured 5 cm in length, whereas the smallest one measured 0.5 cm. The jejunal wall was paper-thin at the gangrenous patches, and the serosa showed the presence of fibrinous exudates. The intervening mucosa was edematous and erythematous. The cut surfaces of the mesenteric vessels did not show any thrombi.

**Fig. 4:**
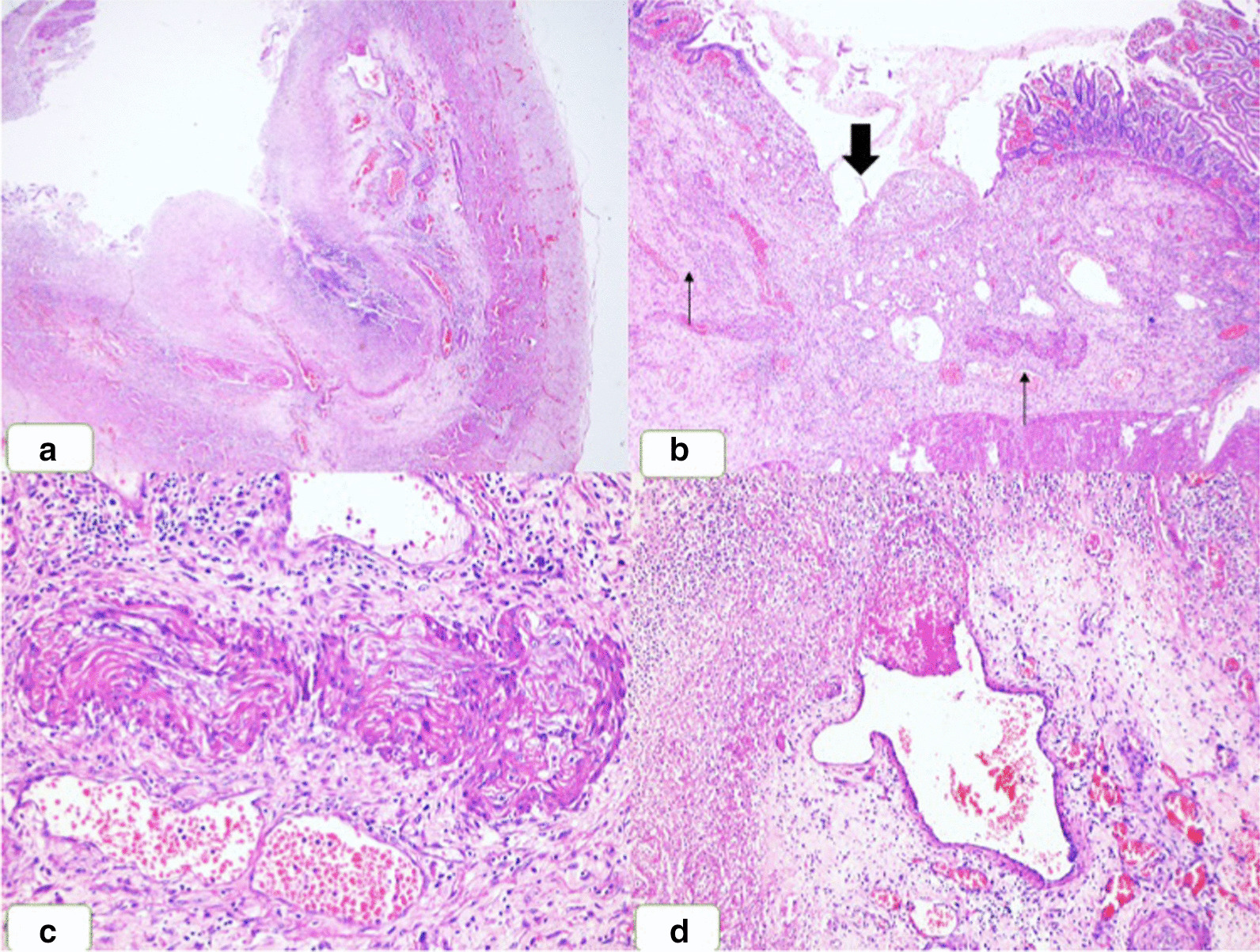
**a** Low magnification showing an intestinal ulcer (hematoxylin and eosin, × 20). **b** Changes in the blood vessels (thin black arrows) highlighted at the base of an ulcer (thick black arrow) (hematoxylin and eosin, × 40). **c** The arteries showing typical changes with luminal occlusion, myxoid subintimal matrix, and hypertortuous eosinophilic internal elastic lamina (hematoxylin and eosin, × 200). **d** One of the veins showing occlusive fibrin-rich thrombus (hematoxylin and eosin, × 100)

Microscopy from different areas showed multiple intestinal ulcers that were superficial mucosal, deep, and transmural (Fig. 4a). The submucosa was fibrotic and contained mixed inflammation. Notably, vascular pathology was demonstrated in both arteries and veins of small and medium caliber at the ulcer base as well as in the submucosal vessels underneath the non-ulcerated mucosa (Fig. 4b). The small and medium-sized arteries showed near-total to complete obliteration by intimal proliferation that showed peculiar fibrocellular hyperplasia in a myxoid stroma. The proliferated intima contained refractile red degenerated elastic material in a few arteries. The corresponding internal elastic laminae of these arteries were extremely hypertrophic, markedly tortuous, and at places crumpled (Fig. 4c). Nevertheless, in a few arteries, the internal elastic lamina showed abrupt discontinuity at places. The arteries did not show any inflammatory infiltrate except for a few where infiltration of the media by a few histiocytes and lymphocytes was noted. The submucosal veins showed a spectrum of changes from containing fresh fibrin thrombi (Fig. 4d) to old occlusive thrombi completely/near-completely obliterating their lumina. The fresh fibrin thrombi were partially occlusive with a cellular inflammatory infiltrate traversing the vessel wall and beyond. Organization and recanalization of these thrombi were also noted. Both the arteries and the veins showed mild adventitial fibrosis. No cholesterol cleft, atheromatous plaque, calcification, or fibrinoid necrosis was noted in any of the vessels to suggest atherosclerosis or other forms of vasculitis. Similar changes were also observed in the resected specimen of the appendix. A diagnosis of thromboangiitis obliterans involving the gastrointestinal tract was rendered.

## Discussion and conclusion

Thromboangiitis obliterans (TAO) is a non-atherosclerotic, vasocclusive disease of young smokers involving small and medium-sized arteries and veins, although about 5% of non-smokers tend to develop the disease [2]. The exclusive male status is also partially offset by a recent rise in its prevalence among the women as a result of their increased smoking activity [2]. The disease typically affects the upper and lower limbs and is one of the common global causes of peripheral vascular disease. The disease is also called “Buerger’s disease” or “Morbus Winiwarter-Buerger” to commemorate Dr. Leo Buerger and Dr. Felix von Winiwarter, the American and Austrian surgeons who are credited with the first descriptions of the disease [3].

The involvement of the gastrointestinal tract is fairly uncommon and that causing mesenteric ischemia is a rarity. The spectrum of clinical manifestations of Buerger's disease of the GI tract may range from chronic intermittent pain of the abdomen, weight loss, chronic diarrhea, to sometimes acute abdomen. The small intestine is the most commonly affected, which is usually segmental. Isolated involvement of the colon and simultaneous involvement of the small and large intestine have also been reported [3]. In most cases, an antecedent history of amputation of some part of one or more extremities was consistent. However, out of 50 cases of Buerger’s disease involving the GI tract that have been reported, 12 had intestinal involvement as an inaugural presentation [4].

Cessation of smoking has a high preventive value. Only 5% of ex-smokers progress to develop the disease in another site following abstinence from smoking in contrast to the 100% progression rate in the patients who continue to smoke [5]. The patient being reported was a known smoker for 20 years, a habit he had quit after he underwent a right midfoot amputation for Buerger’s disease 4 years before. Despite this, he developed acute mesenteric ischemia as well as segmental patchy gangrene and perforation of the small intestine, which makes this case unique. A similar phenomenon was previously reported by Kamiya et al., where the patient had a right below-knee amputation [6].

Acute mesenteric ischemia evolves through four stages, as proposed by Haglund et al. [1]. The first one is the hyperactive stage, which is characterized by intermittent severe abdominal pain, vomiting, and loose stools often associated with blood. The bowel sounds are usually exaggerated in this phase. This is followed by a paralytic stage in which the patient experiences continuous diffuse abdominal pain, abdominal distention, and absent bowel sounds. The stage of deranged fluid balances follows, which leads to leakage of intestinal content into the peritoneal cavity when the patients develop frank peritonitis. If not detected and managed early, these cases progress into a stage of irreversible shock. Hence, the index of suspicion needs to be high in patients presenting with an acute abdomen especially when there is an antecedent history of chronic smoking and amputation involving the extremities. Severe abdominal pain out of proportion to physical findings should be assumed to be in line with acute mesenteric ischemia unless proven otherwise, which is a Level 1b recommendation of the World Society of Emergency Surgery [7]. In our case, the patient presented with non-bilious vomiting and loose stools, which persisted even in the postoperative period after the index laparotomy, which we overlooked. The patient continued to pass blood-mixed stool during the postoperative period, which was a useful clue for suspecting mesenteric ischemia, which was again missed, which proved costly. Eventually, the patient developed fecal discharge from the wound for which he needed to be re-explored. By this time, he had multiple segmental necrotic patches in the jejunum along with two perforations in the ileum, which triggered the possibility of a segmental mesenteric inflammatory/ischemic etiology.

As per the World Society of Emergency Surgery guidelines, CT angiography (CTA) is an investigation modality of choice and should be performed in all highly suspicious cases without delay. It suggests that even deranged renal parameters should not be considered as a contraindication to performing a CT angiography. CTA of the small intestine has a similar appearance to that seen in the extremities. However, the degree of corkscrew appearance is less evident, and characteristically there is involvement of the branches instead of the main trunk [8]. CTA may also demonstrate intestinal dilation, lack of vascularity, pneumatosis intestinalis, and gas in the portal vein [7]. According to a study by Nuzzo et al., bowel loop dilatation is the strongest predictor of intestinal necrosis [9]. However, the index patient did not undergo CTA as Buerger’s disease of the intestine was never suspected. Intraoperative Doppler study can be useful in assessing the vascularity of the bowel segment whenever there is a suspicion irrespective of the color of the bowel (healthy or dusky). Intraoperative palpation of the superior mesenteric artery can be beneficial to rule out thrombosis [7].

Elevated serum lactate > 2 mmol/l is an important predictor of intestinal necrosis [9], and a level > 2.7 mmol/l is a predictor of ICU death [10]. The presence of elevated lactate, organ failure, and bowel dilatation should prompt immediate exploration [9]. Serum D-dimer is not predictive of acute mesenteric ischemia [11]. In this patient, lactate levels were persistently elevated even after multiple explorations, and ultimately the patient developed high anion gap metabolic acidosis.

Intravenous fluids should be administered to improve end-organ perfusion, crystalloids being the fluid of choice [12]. Goal-directed fluid therapy should be carried out with continuous monitoring of hemodynamic parameters and urine output. Supplemental oxygen should be administered immediately to improve the oxygen supply to the ischemic bowel loops [13]. Vasopressors should be avoided as they further reduce the splanchnic blood flow, although vasopressors such as dobutamine and a low dose of dopamine and milrinone can be considered, which have a minimal effect on splanchnic blood flow. Although some studies have advocated the use of heparin in cases of mesenteric ischemia [14], others have reported against it [15]. The ESTES guidelines recommend the use of heparin mostly for mesenteric venous thrombosis and mesenteric arterial embolism. Intestinal mucosal ischemia can lead to translocation of gut bacteria and endotoxins. Therefore, broad-spectrum antibiotics should be initiated although a fixed regimen of choice has not been established [16].

Damage control surgery should be performed if there is a suspicion of mesenteric ischemia, as the viability of suspicious-looking bowel loops can be assessed later, and the status of the anastomotic site can be inspected postoperatively. In our case, the bowel during the first laparotomy was grossly pink and normal looking. An appendicectomy, to which the omentum was adherent, was done before closing the abdomen. However, in the second laparotomy, there were multiple segmental necrotic patches in the jejunum, starting from 5 cm from the DJ flexure through a length of 60 cm of the jejunum. There were two other perforations in the distal ileum adjacent to each other, 10 cm proximal to the ileocecal junction. The necrotic jejunal segment was resected, and Roux-en-Y side-to-side gastrojejunostomy with a hand-sewn side-to-side jejuno-jejunostomy was performed. The ileal perforations were brought out as a loop ileostomy after sending the perforation margins for biopsy. A feeding jejunostomy (FJ) was also performed. The laparotomy wound was again closed with the placement of the abdominal drain, as the possibility of Buerger’s disease of the small bowel was yet to determined in our differentials. In hindsight, a temporary abdominal closure with a Bogota bag or vacuum-assisted closure would have been a better alternative. Vacuum-assisted closure helps in wound healing and also provides an option for a scheduled second-look laparotomy within 48 hours of exploration [12].

A stoma is always preferable to an anastomosis, particularly when the vascularity of the bowel loop is in jeopardy. However, the proximity of the proximal resection margin being very close to the DJ flexure and the concurrent terminal ileal perforation precluded the possibility of a proximal stoma, so we were forced to consider a Roux-en-Y gastrojejunostomy with a jejunojejunostomy along with a distal loop ileostomy. Some cases requiring repeated resection of the intestine have been reported in the literature [17]. Even in our case, a third exploration was required because of leakage from the GJ and FJ sites, which again necessitated fixing the leakage sites, although the mission was not successful.

Histologically, small and medium-caliber arteries and veins are both affected by thrombotic manifestations at variable stages of the process. The acute stage arteriovenous changes are considered pathognomonic with intravascular fibrin thrombi having a centrifugal pattern of inflammatory infiltrate. The subacute changes show the organization of the thrombi with microabscess and/or granuloma and giant cells, a tell-tale feature of Buerger’s disease. The chronic features include recanalization and/or intimal fibroplasia and luminal obliteration [3]. The index case showed a spectrum of arteriovenous changes ranging from acute (fibrin thrombi in the veins with inflammation spreading to the periphery) to subacute (organizing thrombi with inflammation of the arteriovenous wall) to chronic (arterial intimal fibroplasia and luminal obliteration with hypertrophic internal elastic lamina and adventitial fibrosis and recanalizing thrombi in the veins) suggesting a chronic ischemic process with superimposed acute insult.

The role of histopathology in such cases is manifold. First, the vasculopathy is characteristic, although often missed by histopathologists because of its rarity. Also, the history of amputation or Buerger’s disease may be unavailable to the pathologists if clinical suspicion of intestinal Buerger’s disease is lacking. Second, the pathologists play a pivotal role in identifying the stage of vasculopathy, thereby documenting the disease pathogenesis. Lastly, the exclusion of relevant vascular diseases, in particular atherosclerosis and vasculitis, especially polyarteritis nodosa, can be made by histopathological examination.

The strong point of this article is that it highlights the variety of clinical presentations of Buerger’s disease of the intestine and outlines the investigation and management ideally needed. However, as the diagnosis was picked up late in our patient and the patient could not be saved despite multiple surgeries, little could be done to determine the effect of any pharmacological intervention on the management of this condition. However, the prognosis of Buerger’s disease of the intestine is poor as early detection is usually not possible.

A high index of clinical suspicion is crucial for embarking upon early supportive measures. The clinical course can often be deceptive and be further complicated if septicemia developes, auguring a fatal outcome. CT angiography should be done early at the slightest suspicion, especially in a smoker with a prior history of amputation. Exploratory laparotomy and resection of the infarcted/perforated/strictured bowel remain the mainstay of therapy, although single surgery often does not suffice because of the patchy nature of the disease. Despite adequate treatment, a mortality rate of 30% has been reported [3]. The histopathology of the vessels in the resected intestine is typical of this disease.

## Data Availability

Not applicable.

## Abbreviations

TAO: Thromboangiitis obliterans
CTA: CT angiography
ANA: Antinuclear antibody
ANCA: Antineutrophil cytoplasmic antibody
ESTES: European Society of Trauma and Emergency Surgery
DJ: Duodenojejunal
GJ: Gastrojejunostomy
FJ: Feeding jejunostomy
